# Evaluating Cold, Hot, and Social Cognition in Hospitalized Patients

**DOI:** 10.1192/j.eurpsy.2025.1838

**Published:** 2025-08-26

**Authors:** N. Laherran Cantera, R. Palacios-Garran, G. Lahera Forteza

**Affiliations:** 1 Psychiatry, Jerez University Hospital, Jerez; 2 Psychiatry, Principe de Asturias University Hospital, Madrid, Spain

## Abstract

**Introduction:**

Cognitive difficulties in people with psychosis are present at the time of the first episode, even from periods of high risk to early stages of psychosis, their nature and severity seem similar to those observed in patients with a more chronic evolution, since it seems to be an impairment that in most people remains stable, although there is a group of people that worsens. Therefore, these deficits cannot be fully explained by treatments, hospitalizations, or chronicity, and appear more as an intrinsic characteristic of the disease. The course of their trajectory through the progression of the disease remains uncertain.

**Objectives:**

This study aimed to evaluate changes in cold, hot, and social cognition during acute psychotic episodes in hospitalized patients, to better understand their relationship with psychotic symptomatology.

**Methods:**

A prospective longitudinal study was conducted with 10 patients (age range: 18-65) admitted to the psychiatric acute unit at Jerez de la Frontera Hospital, diagnosed with schizophrenia, schizoaffective disorder, or other psychotic disorders. Cognitive assessments included SCIP (cold cognition), Hinting Task (social cognition), and OSCARS (hot cognition), alongside psychiatric evaluations using PANSS, BPRS, and GAF scales. Statistical analysis was performed to identify correlations between cognitive domains and psychotic symptoms.

**Results:**

Analysis of the 10 selected patients reveals that the levels of impairment in cold, warm, and social cognitive functions vary significantly. The mean obtained for the SCIP-S scale total (64.60) suggests a moderate impairment in cold cognition, which aligns with previous research indicating that this type of impairment is an intrinsic feature of psychotic disorders, regardless of the time course of the illness.The mean on the Hinting task (14.00) and OSCAR total score (52.60) reflect impairment in social and warm cognition, respectively. Regarding the PANSS scale, scores indicate a predominance of positive psychotic symptoms, with a mean of 31.50 on PANSS-P, suggesting a high degree of active symptomatology.

These results are congruent with the hypothesis that alterations in warm cognition could precede and perhaps influence the exacerbation of these psychotic symptoms, as has been suggested in previous studies.

**Conclusions:**

This study provides preliminary evidence of the complex relationship between cold, warm, and social cognitive functions in patients with acute-phase psychotic disorder. The findings suggest that although these functions are impaired in psychosis, their interrelationship is not as strong as might be expected, underscoring the need for differentiated interventions that address each cognitive domain specifically.

**Disclosure of Interest:**

None Declared

